# Functional Aging: Integrating Functionality to a Multidimensional Assessment of Healthy Aging

**DOI:** 10.1155/2023/9409918

**Published:** 2023-01-28

**Authors:** Emilia Frangos, Christophe Graf, Nikolaos Samaras

**Affiliations:** ^1^Division of Internal Medicine and Rehabilitation Loëx—Joli-Mont, Department of Rehabilitation and Geriatrics, Geneva University Hospitals, Geneva 1211, Switzerland; ^2^Division of Geriatrics, Department of Rehabilitation and Geriatrics, Geneva University Hospitals, Geneva 1211, Switzerland

## Abstract

Worldwide, the number of old adults will peak in the coming decades. Relying solely on the chronological age to make treatment decisions and shape general or specific societal and medical considerations may reinforce ageism and lead to flawed reasoning. Defining physiological age using biological markers is not yet reliable, and an approach based on comorbidities without considering their impact on quality of life is inadequate. A multidimensional approach with strong integration of functionality is presented here to draw a real-world aging approach, easily accessible, clinically relevant, and of societal value.

## 1. Introduction

Increased life expectancy all around the world, together with the decline in birth rates, contributes to a rising proportion of older people. Between 2015 and 2050, the proportion of people over 60 years old will increase from 12% to 22% to reach 1.5 billion people. Projections show a clear doubling of this proportion between 2000 and 2050 [1]. The number of oldest old persons (more than 80 years old) will reach 395 million in 2050 [1].

The studies, reasonings, decisions on treatments and investigations, as well as many general or specific medical considerations, are based on patients' chronological age, with an approach that can be common for patients of the same age (or patients of different ages of 65-year-old adults and over centenarians included in the same group), but in completely different conditions.

*Biological age* is directly linked to progressive and random deteriorations which occur at multiple levels and alter the functions of organs, but its definition needs multiple, specific, and complementary biomarkers. Otherwise, the aging population can be considered based on *comorbidities*, but with the risk of misevaluating their impact in everyday living, which can be very variable in terms of induced disability. Therefore, how to reach the picture of so-called *healthy aging*? Here, we aim to highlight the advantages, particularly in the qualitative assessment of the healthy aging process, of the multidimensional approach integrating *functionality* proposed by the WHO [2].

### 1.1. Aging and Classical Biomarkers of Age

*Aging* was defined by the WHO as the result of the accumulation of a wide variety of molecular and cellular degenerations over time, leading to a gradual decline in overall physical and mental health [3]. Dahlgren and Whitehead propose a model with several *determinants of health* such as age, sex, constitutional factors, individual lifestyle factors, social and community networks, and general socioeconomic, cultural, and environmental conditions [4]. That is to say that the issues of aging are multiple and complex.

A systematic review in 2020 showed that in 85% (*N* = 127) of 149 studies, the age determined who received certain medical procedures or treatments [5]. Evidence of ageism was found in all 49 studies that investigated the link between age and exclusion from different types of health research, older persons being systematically excluded from clinical trials in internal medicine, and several medical specialties, even though many of the conditions under study are more prevalent in older age [5, 6]. There is a high risk of ageism (referring to stereotypes, prejudice, and discrimination towards others or oneself based on age) if chronological age remains the main and/or only consideration [7].

As age-related healthcare rationing is already widespread [7], it is important to search for other ways to more objectively appreciate the speed of aging, predict potential longevity, screen diseases in a population, ease diagnosis at an individual level, as well as estimate the response to medical treatment and relapse after treatment [8]. Indeed, the hallmarks of aging, which include genomic instability, telomere attrition, epigenetic alterations, loss of proteostasis, deregulated nutrient sensing, mitochondrial dysfunction, cellular senescence, stem cell exhaustion, altered intercellular communication, compromised autophagy, microbiome disturbance, altered mechanical properties, splicing dysregulation, and inflammation appear to be an essential basis to evaluate biological age [9, 10]. DNA methylation changes [11] have been studied in combination with external risk factors (smoking, alcohol consumption, physical activity, chronic diseases, and body mass index) (GrimAge) [12]. Those referent technics, which show their relevant associations with several clinical outcomes, notably in terms of mortality and disease manifestations, are currently used mainly in research.

In parallel with these biological hallmarks of aging, other markers are still investigated in longitudinal studies such as anthropometric markers (e.g., height, weight, BMI, or abdominal circumference), biochemical factors (e.g., hemoglobin, glycated hemoglobin, cholesterol, albumin, CRP, cytokines, inflammation factors, and chemokines), or physiological markers (blood pressure, lung function, or glomerular filtration), in an attempt to capture physiological changes along the aging courses and to assess health risks in individuals of the same age [11, 13, 14].

However, as there are a lot of different aging pathways and processes [15], the results are unreliable [16], and these different approaches of aging do not allow for drawing by themselves a precise “clinical” picture of old people concerning their capacities in daily activities, which are essential for the concept of *functionality* in geriatric medicine.

### 1.2. Functional Aging

In 1948, the WHO defined “good health” in its constitution as a state of complete physical, mental, and social well-being and not merely an absence of disease or infirmity [17]. In 2015, the WHO report on Aging and Health built the definition of “healthy aging” on the notion of functional ability, rather than the mere presence or absence of disease. “Healthy aging” is now considered “the process of developing and maintaining functional ability which enables well-being in older age” [2, 18].

*Functionality* is defined as “the quality of being suited to serve well” (Oxford Language) the state of being functional or having a good level of body function. The ability to execute a task and participate in life activities fully matches this concept. On the contrary, disability is defined as “a difficulty or dependency in carrying out activities essential to independent *(functional)* living, including essential roles, tasks needed for self-care, and living independently in a home, and desired activities important to one's quality of life” [19]. Disability can contribute to *dependence* when it cannot be reduced by technical aids (e.g., glasses or hearing aids for sensory deficits) [20]. Therefore, defining a *functional age* (or functional capacity during the aging process) of an old person fully correlates not only with the definition of “healthy aging” but also with the relevant notion of quality of life and *activity* in daily living.

“*Activity*” has a leading and central position in the WHO International Classification of functioning, disability, and health model [20]. “Activity” may be influenced by clinical health conditions (coexistence of two or more health conditions often increases exponentially the number of impairments), participation abilities, body functions (reduction of muscle strength in both upper and lower limbs and changes in body-fat percentage, flexibility, agility, and endurance), and contextual (environmental and personal) factors [20–22]. Therefore, a functional assessment of older persons appears essential and has been proven to better assess physiological age in longevity studies, in combination with the biological assessment [11]. Moreover, it has been proven far more useful than merely focusing on or simply measuring chronological age. For old adults themselves, the main elements that define the perceived variability in one's health are of course health problems (physical health, symptoms, and diseases), health habits (nutrition, physical exercise, etc.), state of mind, but also physical functionality (activity, physical, or sensory disabilities) [23]. Quality of life is also influenced if self-rated by functional status [24] and self-reported physical fitness evaluated by specific scales [25]. Physical fitness is related to activity and its basis in old age is a sufficient level of physical and motor independence [26].

Physical abilities tend to decline with age, but it is not a fatality. In Japan, where the older population 65 years of age and above reach almost 30%, a recent study shows that based on six indices related to health and functioning (height, weight, body mass index, walking speed, grip strength, and instrumental activities of daily living), the health status was better and the decline in most of the measures was slower in a 2017' cohort compared to a decade ago [27]. However, when mobility loss results from multiple impairments in the central and peripheral nervous system, muscles, joints, energetic and sensory physiological systems, already at an early preclinical stage, it interferes with daily life activities and independencies [27]. In fact, in healthy mid-life adults, functional trajectories start to decline around 40–50 years old in men and 50–60 years old in women, as described for walking speed [28] or open-eyes one-leg stand [29]. This “silent” lowering of functional supply contributes to the definition of *functional aging.* Before describing its relevant clinical significance in terms of outcomes, we will overview its assessment.

### 1.3. Functional Assessment

Being “functional” is not being “disabled” and being able to live, activate, and care independently, as defined by Fried [19]. Functionality can be assessed by different methods, through a “physical” or a more “cognitive” approach. As the two are often intricate, a global approach is primarily recommended, with an assessment of the ability to perform in an autonomous way basic activities of daily living (ADL): bathing, dressing, toileting, transferring, continence, and feeding [30]. In addition, the ability to perform independently instrumental activities of daily living (IADL) can be assessed as follows: the ability to use the telephone, do shopping, prepare food, do laundry, use transportation, be responsible for own medications, and able to handle finances and housekeeping [31].

Another functional global assessment tool, more practical and useful in older people, especially in hospitals, is the functional independence measure (FIM) which assesses 18 daily activities separated into two groups (motor or cognitive items), each one including six different domains: four in the motor group (self-care, sphincter control, mobility, and locomotion) and two in the cognitive group (communication-social interaction and cognition). Each item is scored from 1 to 7, in accordance with the older person's abilities, from major functional impairment to total independent functioning for the tested activities. The total observational scores vary linearly from 7 (total dependence) to 126 (total independence) [32–35].

In addition to these global functionality assessment tools, others permit to evaluate the basic motor performance of older adults, by measuring strength in the upper and lower limbs (handgrip strength or leg extension and flexion), time needed to get up and go [36], ability to stand up from a chair, and walking speed or 6-minute walking test [27, 37]. The Short Physical Performance Battery (SPPB) assesses lower-extremity function according to three parameters: standing balance, walking speed and ability, and a timed test of five times rising from and sitting in a chair [38, 39].

The results of these mobility-functional tests have been associated with functional limitations directly related to ADL and IADL. For example, it was proven that the grip test evaluates global health as it is associated with a risk of malnutrition, osteoporosis, sarcopenia, falls, and depression/dementia [40, 41]. An altered chair rise test alarms on bathing, safety, toilet transfer, housework, etc. Physical endurance assessed by walking tests raised suspicion against community mobility and shopping difficulties.

In epidemiological studies of older people, the PASE (Physical Activity Scale for the Elderly) has been used as a practical score to assess the frequency, duration, and intensity level of activity over the previous week, with combined information on leisure time and household and occupational activity [42]. The SFTB (Senior Fitness Test Battery) is another test to assess strength (chair stand test for lower and arm curl test for upper body), flexibility (chair sit and reach test for lower and back scratch test for upper body), agility (8-foot up and go test), and aerobic fitness (walk or step in place test) [43].

Evaluation of other aspects of functionality, such as sensory and cognitive function can be conducted through the well-known Snellen chart, whisper test, geriatric depression and anxiety screening, and specific tests for memory loss or the clock drawing test [21].

### 1.4. Function Tests as Markers of Poor Clinical Outcomes and Mortality

The functional status is closely interrelated to comorbidities and chronic diseases, often contributing to physical limitations [19]. The main comorbidities responsible for functional limitations are also the most prevalent in older populations: osteoarthritis, diabetes, cardiac/neurological vascular pathologies, chronic obstructive pulmonary diseases, and sensory impairments, as well as depression and various musculoskeletal disorders [44]. In those aged over 80, dementia is a major cause of disability [45].

Therefore, closely entangled comorbidities and functionalities (evaluated by FIM) were compared as predictive factors of mortality in a hospitalized old population during a 6-year follow-up. After adjustment for age and gender, the number of diagnoses was related to death variability. In comparison, the FIM score accounted on its own for a 14-fold higher risk of death, demonstrating that daily functioning evaluated on a global scale was much more valuable in survival prediction than the number of medical diagnoses [35]. ADL and IADL were also independent predictive factors of intrahospital mortality, as well as predictors of delirium, nosocomial infections, and longer hospitalization [46]. In a community-dwelling older population, handgrip strength and TUG tests were independent and strong predictors of short-term mortality [47]. Low scores in the 6-minute walking test, low-walking speed, and low scores in the SPPB have also been associated with mortality [48]. Additionally, general gait speed [49] and low limb muscle strength measured by the chair stand test [50] were predictors of mortality. In older patients, walking speed is also known to be a predictive factor of overall function in daily living, depressive state, risk of falling, deteriorated ADL, and admission to a nursing home [27, 51, 52]. An SPPB score lower than 10 is predictive of all-cause mortality [53], risk of ADL impairment, falls, and mobility impairment [54]. The SPPB as well as walking speed are associated with a higher risk for future cognitive decline [55, 56].

Concerning grip strength, evidence is also provided for a predictive link with mortality, future function, bone mineral density, fractures, cognition and depression, and nosocomial complications. Therefore, it can be used for identifying older adults at risk of poor health status [41, 57].

Regarding the risk of nursing home admission, functional impairment is of course a strong predictor. This was not the case for several other severe comorbidities studied (stroke, high blood pressure, respiratory diseases, urinary incontinence, or depression) [58]. A high level of functionality is certainly associated with a higher quality of life in the old population and better perspectives for healthy aging [59]. In a recent review of various biomarkers of aging, the practicability of physical function assessment and anthropometry markers' measurement was clearly defined as higher than blood-based molecular/DNA or other novel markers. Moreover, physical function markers were associated with a large range of outcome predictions as described [60].

Finally, a recent and large 20-year longitudinal study of 845 adults with 3973 repeated biological age measurements was examined in terms of correlation with chronological age and mortality [11]. It appears that different types of specific functional measurements are collected to create composite scores, or “indices” which are extremely valuable compared to or in association with DNA methylation scores. It is the case of the functional aging index (FAI-including self-reported vision and hearing sensory ability, combined with muscle strength, walking speed, and lung function recorded by nurses) or the frailty Index (FI-constructed from self-reported health deficits such as symptoms, diseases, disability, mood, and activities of daily living, and calculated as a ratio of deficits for a person to a total number of 42 deficits) [11].

### 1.5. Functional Health

As described, poor functionality is associated with unfavorable clinical outcomes and mortality. Healthy 75-year-old men are estimated to remain in good health for 4.40 years (90.3%) of their 4.87 years of expected total life remaining and 75-year-old women for 4.60 (93.3%) out of 4.93 years, in Australia and the USA [61]. Therefore, it is essential to consider the number of years a person lives without a disability, even if we know that the aging process is related to an ever-growing risk of disease.

The prevention of functional decline *by promoting healthy functional aging* is a major public health challenge. The functional health of today's oldest-old with reported ADL is better than in the past, among women in particular [62] The prevalence of old age dependency has decreased, because of advances in both healthier lifestyles, medical and rehabilitative care, as well as better socioeconomic factors and innovations in a mainstream and assistive technology [63, 64].

All need to be conscious of their own individual responsibility in the aging process. Reducing long periods of disability is a key to health while aging [45], in the WHO, described the process of developing and maintaining *functional ability* which “enables well-being in older age [18].” Thus, there is a great need of revisiting our care policies and programs by laying greater emphasis on the *functional aging* of the population throughout life, focusing on functional detection, assessment, and prevention, instead of focusing on chronological or biological age only.

## 2. Conclusion

A medical approach based on chronological age alone carries a high risk of ageism for geriatric patients. The biological approach can be complicated and not always relevant to define healthy aging. Defining it only through comorbidities is reductive notwithstanding that they are interrelated with functionality since there is a high risk of misevaluation of their impact on everyday living abilities. The functional approach as presented here is widely accessible, clinically relevant, easy to apply, adapted to the older population, and associated with clinical outcomes and of societal value. Strongly integrating this approach to a global assessment including other markers and comorbidities allows a multidimensional definition of healthy aging.

## Data Availability

Data for this article are not provided
